# Human reliability analysis of high-temperature molten metal operation based on fuzzy CREAM and Bayesian network

**DOI:** 10.1371/journal.pone.0254861

**Published:** 2021-08-02

**Authors:** Yaju Wu, Kaili Xu, Ruojun Wang, Xiaohu Xu

**Affiliations:** 1 School of Resources and Civil Engineering, Northeastern University, Shenyang, PR China; 2 School of Safety Engineering, Shenyang Aerospace University, Shenyang, PR China; Al Mansour University College-Baghdad-Iraq, IRAQ

## Abstract

Human errors are considered to be the main causation factors of high-temperature molten metal accidents in metallurgical enterprises. The complex working environment of high- temperature molten metal in metallurgical enterprises has an important influence on the reliability of human behavior. A review of current human reliability techniques confirms that there is a lack of quantitative analysis of human errors in high-temperature molten metal operating environments. In this paper, a model was proposed to support the human reliability analysis of high-temperature molten metal operation in the metallurgy industry based on cognitive reliability and error analysis method (CREAM), fuzzy logic theory, and Bayesian network (BN). The comprehensive rules of common performance conditions in conventional CREAM approach were provided to evaluate various conditions for high-temperature molten metal operation in the metallurgy industry. This study adopted fuzzy CREAM to consider the uncertainties and used the BN to determine the control mode and calculate human error probability (HEP). The HEP for workers involved in high-temperature melting in steelmaking production process was calculated in a case with 13 operators being engaged in different high-temperature molten metal operations. The human error probability of two operators with different control modes was compared with the calculation result of basic CREAM, and the result showed that the method proposed in this paper is validated. This paper quantified point values of human error probability in high-temperature molten metal operation for the first time, which can be used as input in the risk evaluation of metallurgical industry.

## 1. Introduction

Metallurgical enterprises are the most important basic industries in the national economy. After technical transformation and upgrading, the intrinsic safety level of equipment and facilities in metal smelting enterprises has been significantly improved. However, accidents still occur from time to time in the process of metal smelting. According to the statistical analysis of accidents in 36 large iron and steel enterprises reported by the Metallurgical Safety Committee of China Safety Association, in 2015, there were a total of 467 casualties in 36 large iron and steel enterprises, including 29 deaths, 17 serious injuries and 421 minor injuries. The number of casualties was 479, among which 35 were killed, 19 were seriously injured, and 425 were slightly injured [1]. It is also pointed out that the high-temperature molten metal is still the focus of monitoring in the process of metal smelting and production, and it is a major risk factor which is prone to mass fatalities and mass injuries. Statistics show that from 2010 to 2013, 31% of accidents in metallurgical enterprises are related to high-temperature molten metal [2]. In 2015, the most common cause of casualties in iron and steel enterprises is still violation of operating procedures or labor discipline [3], causing 23 deaths, 7 serious injuries and 7 minor injuries, respectively accounting for 60.52% of the total number of deaths, 58.33% of the total number of serious injuries and 62.71% of the total number of casualties. Therefore, human factors are assumed to be the main causation factors in metallurgy accidents, metallurgical industry complex work environment and, especially, high-temperature molten metal work conditions have important effects on human behaviour, causing human error problems, but the metallurgy industry workers lack reliability data and also limits human reliability research. Therefore, it is very necessary and meaningful to study the reliability analysis method for evaluating human error in high temperature molten metal operation.

The human reliability analysis method (HRA) was developed two generations ago to study human reliability in a systematic manner. Cognitive reliability and Error Analysis Method (CREAM) is a representative method of the second-generation HRA method [4], which relies on the contextual control model (COCOM). In addition, it emphasizes the significant effects of the work environment on human behaviour [5].

In recent years, CREAM has been widely used in light of its advantages, emphasizing the important influence of the work environment on human reliability. He et al. [6] proposed a simplified CREAM quantitative analysis method and applied it to the human error analysis of SGTR events in Qinshan No. 1 Nuclear Power Plant. Ribeiro et al. [7] presented a human reliability analysis model based on THERP–CREAM.In addition, CREAM has also been used in the HRA of a liquified natural gas(LNG) terminal in a power supply system [8] and oil tanker ship operation in the maritime industry [9]. Wu et al. [10] proposed a modified cognitive reliability and error analysis method (CREAM) for estimating the human error probability in the maritime accident process on the basis of an evidential reasoning approach.

However, human reliability analysis, especially for the work environment of high- temperature molten metal operation, is rarely involved. Therefore, this paper introduces CREAM to analyze the human reliability of high-temperature molten metal operation. Human behavior is influenced by the working environment, and there is a certain relationship between the factors of the working environment [11]. In CREAM, the factors that introduced to evaluate and decide the Contextual Control Model (COCOM) are called Common Performance Condition (CPC). The determination of the Common Performance Condition depends on the on-site observation and acquisition of professionals. The information provided is characterized by ambiguity, incompleteness and other uncertainties [12, 13]. Fuzzy set theory plays an important role in human reliability analysis [14]. In many references quoting CREAM, fuzzy logic is well applied to deal with uncertain information [8, 15–17]. Zhang et al. [8] introduced fuzzy logic in order to increase the accuracy of CREAM.Chen et al. [17] introduced the interval type-2 fuzzy sets to describe the uncertainty of experts’ evaluation of CPC, and used ANP method to determine the weight [18].

In addition, in the traditional CREAM, the precise/point human error probability cannot be given, and there is an interdependent relationship between CPC, so some literature introduce Bayesian network analysis under the condition of analyzing human error. Kim et al. (2006) [19] put forward the application of Bayesian network to determine the human behavior control mode of CREAM, and get the best estimate of the control mode given the available data and information about the context. Yang Z L. (2013) [20] applied the combination of Bayesian network and CERAM to analyze human reliability in offshore engineering. Golestani N et al. [21] used hierarchical Bayesian networks to explain the causal relationships between environmental factors, human error patterns, and scenario-based activities. Chen et al. [22] utilized CREAM algorithm to calculate the prior probability of each root node error on the basis of constructing the Bayesian network in the diving process.

Human error is an important cause of accidents in high-temperature molten metal operation. The complex environment in which metallurgical enterprises operate on high-temperature molten metal has an important influence on human behavior. At present, there is still a gap in the study of human error in high-temperature molten metal operation, which will provide reference for the safety training of high-temperature molten metal operation, and also provide data input for quantitative risk assessment of metallurgical enterprises [23–27]. Therefore, this paper aims to quantify the human error in high temperature molten metal operation.

According to the discussion above, this paper proposes a human error quantification method of high-temperature molten metal operation, which incorporates CREAM, fuzzy logic theory and Bayesian network method. This model can provide accurate value of human error probability instead of interval value, and can also reflect the relationship between multiple factors. Therefore, the contributions of this paper can be summarized as follows:

1. Firstly, based on the CREAM method, this paper proposes the assessment rules of Common Performance Condition(CPC) suitable for high temperature molten metal operation, considering the characteristics of high temperature molten metal operation environment.
2. Besides, this paper combines CREAM, fuzzy set theory and Bayesian network method to provide the precise/point human error probability (instead of interval value) of different positions of high temperature molten metal for the first time.

The rest of this paper is structured as follows. In Section 2, based on CREAM, the assessment rules of CPC are proposed according to the conditions in high-temperature molten metal operation. Section 3 presents our research methodology. In Section 4, a case study with gaunpersonnel scenarios in different high-temperature molten metal operations is presented to demonstrate the applicability of the proposed HRA model. Section 5 draws the conclusions.

## 2. Cognitive reliability and error analysis method

CREAM emphasizes the significant influence of the context on human reliability. Nine CPCs are introduced to evaluate and decide the COCOM [28–30], as listed in Table 1. The HEP interval of each COCOM is shown in Table 2.

**Table 1:** CPCs and their effects on human reliability.

CPC name	Level	Effect on reliability
1. Adequacy of organisation	Very efficient	Improved (+1)
	Efficient	Not significant (0)
	Efficient	Reduced (-1)
	Deficient	Reduced (-1)
2. Working condition	Advantageous	Improved (+1)
	Compatible	Not significant (0)
	Incompatible	Reduced (−1)
3. Adequacy of man machine interface (MMI) and operational support	Supportive	Improved (+1)
	Adequate	Not significant (0)
	Tolerable	Not significant (0)
	Inappropriate	Reduced (−1)
4. Availability of procedures/plans	Appropriate	Improved (+1)
	Acceptable	Not significant (0)
	Inappropriate	Reduced (−1)
5. Number of simultaneous goals	Fewer than capacity	Not significant (0)
	Matching current capacity	Not significant (0)
	More than capacity	Reduced (−1)
6. Available time	Adequate	Improved (+1)
	Temporarily inadequate	Not significant (0)
	Continuously inadequate	Reduced (−1)
7. Time of day	Day	Not significant (0)
	Evening	Reduced (−1)
	Night	Reduced (−1)
8. Adequacy of training and expertise	Adequate high experience	Improved (+1)
	Adequate, limited experience	Not significant (0)
	Inadequate	Reduced (−1)
9. Crew collaboration quality	Very efficient	Improved (+1)
	Efficient	Not significant (0)
	Inefficient	Not significant (0)
	Deficient	Reduced (−1)

**Table 2:** HEP interval of each COCOM.

HEP interval	COCOM
0.00005 < p < 0.01	Strategic
0.001 < p < 0.1	Tactical
0.01 < p < 0.5	Opportunistic
0.1 < p < 1.0	Scrambled

**Fig 1:**
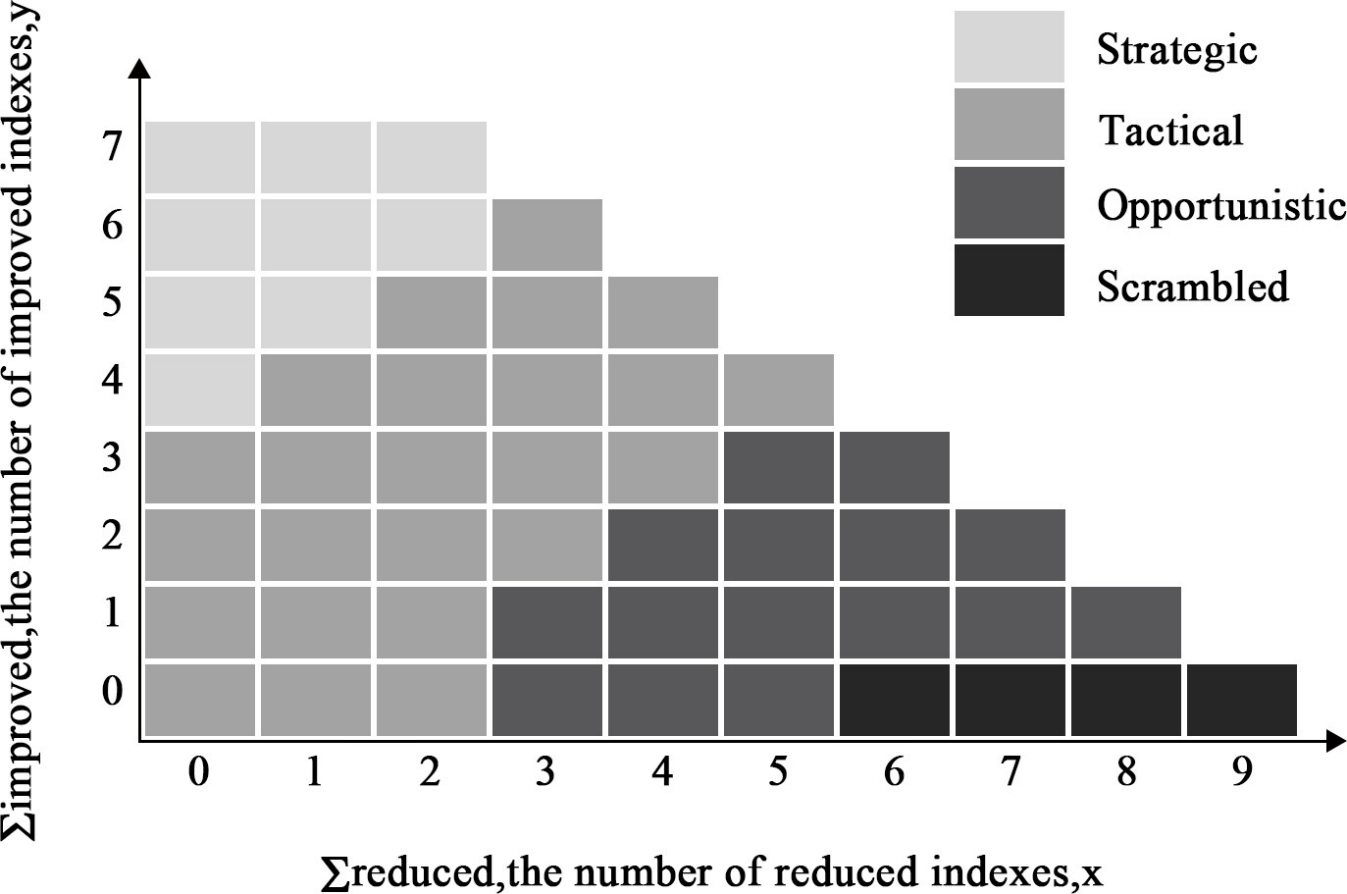
Relations between original CPC score and control mode (Hollnagel, 1998).

The control mode that the operator is likely to use is defined by the arithmetic sums of positive and negative influences of the CPCs (see Fig 1). For the given situation, the description of CPCs results in a specific value for the combined CPC. Subsequently, the control mode for the situation is obtained. According to the original CPCs in Fig 1, if five CPCs affect human performance negatively, three CPCs affect positively, and the remaining CPCs cause neutral effects, then x = 5 and y = 3. The value (x, y) = (5, 3) will be located at the control mode of opportunistic COCOM, and the value of HEP will be between 0.01 and 0.5, as seen in Table 2. This study proposed new CPC assessment rules for high-temperature molten metal process. Each CPC contains several sub-influence factors that are shown in S1 Appendix.

## 3. Research methodology

### 3.1 Fuzzy model

#### 3.1.1 CPC level based on fuzzy model (level)

Subjectivity is a major flaw in HRA. Meanwhile, due to the lack of HEP data and the fuzzy characteristic of CPC at various levels in CREAM, fuzzy logic has been widely applied to deal with uncertain information.In Table 1, each CPC has multiple levels and each level has a different effect on human reliability. The symbols ‘+1’, ‘0’, and ‘-1’ represent positive, neutral, and negative impacts on human reliability performance, respectively. When CREAM is used, various CPC levels are described according to the operating environment, and the expected effects on human performance reliability are determined. The evaluation of CPC is expressed by a value between 0 and 100 and converted to fuzzy output. The purpose is to transform discrete language variables into fuzzy sets. Membership functions of each CPC level language variable need to be established. Triangle and trapezoid membership functions are the most commonly used. We used the trapezoid membership function *f(x)*, which is written as follows:

$$f\left(x\right)=\{\begin{array}{ll} 0 & \left(\text{x}\leq \text{a}\right)\\ \frac{x-a}{b-a} & \left(\text{a}\leq \text{x}\leq \text{b}\right)\\ 1 & \left(\text{b}\leq \text{x}\leq \text{c}\right)\\ \frac{d-x}{d-c} & \left(\text{c}\leq \text{x}\leq \text{d}\right)\\ 0 & \left(\text{x}\geq \text{d}\right) \end{array},$$ (1)

where *x* is in the range of 0 to 100, or calculated using the logarithm of HEP data.

#### 3.1.2 Defuzzification

**Fig 2:**
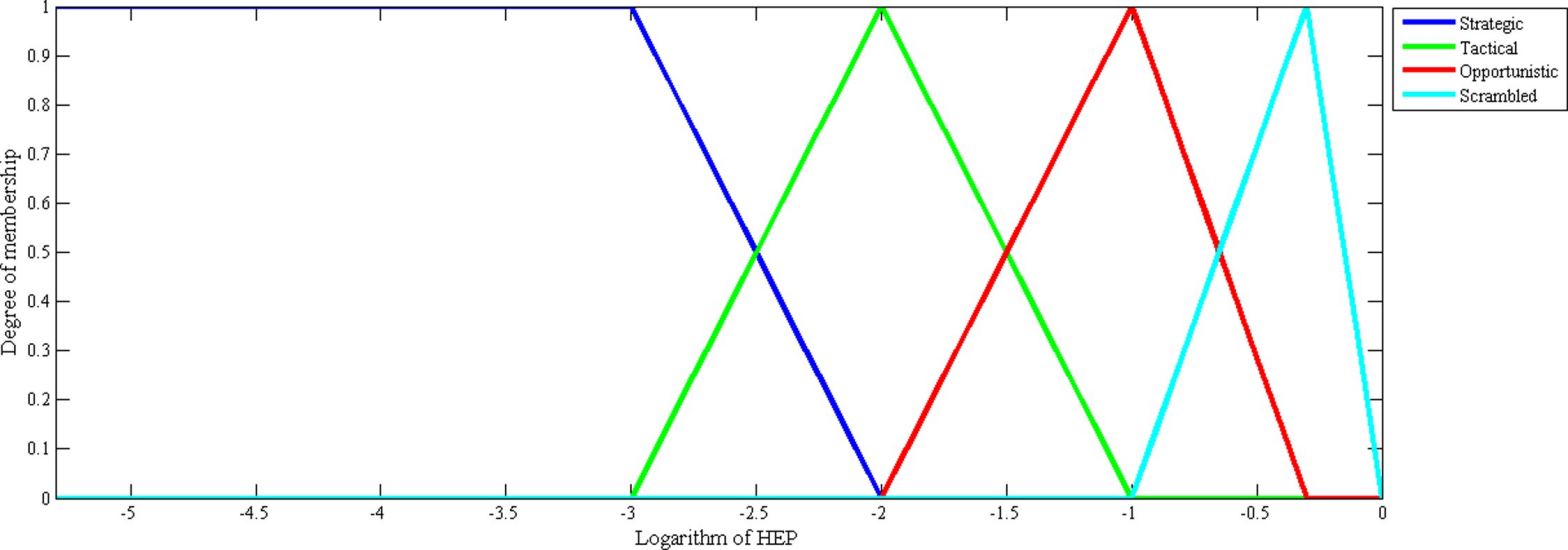
Membership curves of each COCOM.

With fuzzy membership data for each CPC, the membership degree of each control mode can be determined; however, because it is not a definite value, it cannot be used practically. Defuzzification is to transfer the fuzzy conclusions to a crisp value, which is the logarithmic value of HEP. Defuzzification methods include centre of area (COA), maxima, mean of maxima, weighted mean of maximums, and centre average weighting methods [15, 31]. Subsequently, COA was adopted for calculating the HEP result, as shown in Eq (2).

$$\log_{10}HEP=\frac{\int_{X}xf\left(x\right)dx}{\int_{X}f\left(x\right)dx}$$ (2)

where *f(x)* is the aggregated membership function for control modes, which can be calculated by Eq (3) and the curves in Fig 2.


$$f\left(x\right)=f_{str}\left(x\right)+f_{tac}\left(x\right)+f_{opp}\left(x\right)+f_{scr}\left(x\right)$$ (3)

### 3.2 CPC adjustment based on BN

The control mode can be immediately determined using Table 1 and Fig 1 to calculate the HEP interval. However, it neither provides a precise probability value nor considers the interrelationships among CPC’s. Therefore, this paper employed the BN method in the analysis of CREAM.

The BN proposed by Pearl by summarizing a previous work is a directed network that described probability relations based on probabilistic reasoning [20] It has been widely used for system modelling, reasoning, diagnosing, and risk assessment purposes [32–34]. The BN is a probabilistic graphical model that represents a set of random variables and their conditional dependencies via a directed acyclic graph (DAG) with nodes and arcs, where the nodes represent random variables and the arcs represent the probabilistic dependencies between the variables. This is mainly a tool allowing the analyst to exploit different information in a deterministic or probabilistic emerging from the real world, under the condition of complex relations between many variables [3, 4].

Consider a DAG (Laijun Zhao, 2012), *N = (V*, *E*), where V denotes a set of nodes and E denotes a set of directed edges. We can describe a joint probability distribution P over a set of variables *V* = {*X*_1_,*X*_2_,⋯,*X*_*n*_,}, which can be factorized as follows:

$$P_{i}(X_{1},X_{2},\cdots ,{\text{X}}_{\text{n}})=\prod_{\text{i=}1}^{\text{n}}\text{P}\left({\text{X}}_{\text{i}}|Parent\left(X_{i}\right)\right)$$ (4)

where *Parent*(*X*_*i*_) is the set of parent variables of variable *X*_*i*_ for each node *v* ∈ *V*. The nodes in V are in one-to-one correspondence with the variables *X*_*i*_. For a node without any parent nodes, the conditional probability is identical to the prior probability.

The CPCs of CREAM due to their effects on human performance reliability may depend on each other. Then, a CPC needs to be adjusted first and the relationship between the CPCs and control modes can be modelled realistically and systemically in COCOM using the BN.

The rules were used to determine the degree of dependency among the CPCs. Table 3 shows such dependencies. In Table 3, CPC2, 5, 6, and 9 are adjusted (principal) CPCs and CPC1, 3, 4, 7, and 8 are unadjusted (dependent) CPCs.

**Table 3:** Rules for adjusting CPCs.

Principal CPC	Dependent CPCs				
**Adjust CPC2(4/5)**	CPC1	CPC3	CPC6	CPC7	CPC8
**Adjust CPC5(2/3)**	CPC2	CPC3	CPC4		
**Adjust CPC6(4/5)**	CPC2	CPC3	CPC4	CPC5	CPC7
**adjust tCPC9(2/2)**	CPC1	CPC8

1. Adjustment rule of CPC2: If four CPCs out of CPC1, CPC3, CPC6, CPC7, and CPC8 have an effect of ‘improving’ / ‘decreasing’ on human reliability, then the effect of CPC2 on human reliability is adjusted to ‘improving’ / ‘decreasing’.
2. Adjustment rule of CPC5: If two CPCs out of CPC2, CPC3, and CPC4 have an effect of ‘improving’ / ‘reducing’ on human reliability, then the effect of CPC5 on human reliability is adjusted to ‘improving’ / ‘reducing’.
3. Adjustment rule of CPC6: If four CPCs out of CPC2, CPC3, CPC4, CPC5, and CPC7 have an effect of ‘improving’ / ‘reducing’ on human reliability, then the effect of CPC6 on human reliability is adjusted to ‘improving’ / ‘reducing’.
4. Adjustment rule of CPC9: If the influence of CPC1 and CPC8 on human reliability is ‘improving’ / ‘reducing’, then the influence of CPC9 on human reliability is adjusted to ‘improving’ / ‘reducing’.

In CREAM, if a BN is directly established according to its nine CPC factors, 31,104 (43 × 35 × 2) conditional probabilities were assessed to build a network, which is an extremely difficult task. There is one way for reducing the required conditional probabilities, which is to reduce the number of parent nodes using a divorce approach. The CPCs are classified in three groups based on the second approach:

G1: CPC1, adjust CPC2, CPC3
G2: CPC4, adjust CPC5, adjust CPC6
G3: CPC7, CPC8, adjust CPC9

This classification does not have any physical meaning and is used only to reduce the software load.

**Fig 3:**
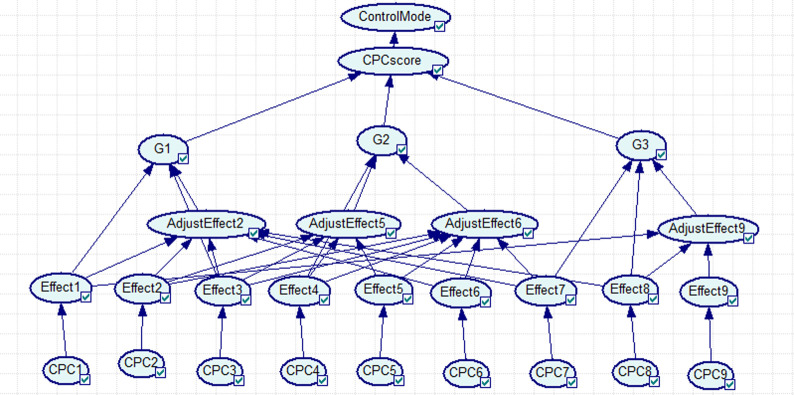
Using the BN for determining the probability distribution of the control modes.

Therefore, Fig 3 is the BN of CREAM control model, which is appropriate for determining human reliability of processing high-temperature molten metal. According to Fig 3, the first layer is the input layer, and the fifth layer in the network is the control mode output layer.

## 4. Case study

Our proposed methodology was used to analyse the human reliability of different operating procedures of high-temperature molten metal in a Chinese steelmaking enterprise.

### 4.1 Ethics statement

The study protocol was approved by the Human Subjects Ethics Sub-committee of Northeastern University. All participants agreed to participate in the study. The HSE department of Benxi Beiying Steel (Group) Co., Ltd. provided the basic data for risk assessment in the paper. The 13 operators mentioned in the study have been informed, and agreed to participate by oral consent and to be evaluated. Additionally, we state that the 13 operators were only observed during their work.

### 4.2 Data input

In the process of steelmaking production involving high-temperature melting, erroneous operations of an operator are listed in Table 4.

**Table 4:** Ranks, human erroneous operations, and cause accidents at different positions.

No.	Position
1	Torpedo tankers transport molten iron
2	Scrap steel workers
3	Shaker mixing molten iron
4	Desulfurization injection
5	Desulfurization and slag removal operation
6	Converter blowing operation of the steel-making master
7	Crane operator for lifting of molten steel water tank
8	Hoisting crane operator who confirms that the hook is in place
9	Tundish repairman
10	Operator and controller of oxygen lance for steelmaking
11	Temperature measurement and sampling operator
12	Slag dumping operation of shaker
13	Argon blowing operator of converter

In the operational processes listed in Table 4, accidents may be caused by human mistakes. For example, the operation is not standardized and the station is wrong.Experienced metallurgical industry experts were asked to rate the CPC factors involved in the above processes. The CPC evaluation rules are shown in S1 Appendix with a maximum score of 100. In addition, considering industrial experiences (a number of metallurgical enterprises were evaluated), working hours, and technical titles, the weights of four experts were obtained as 0.3, 0.26, 0.24, and 0.2. After considering the weights of experts, the final score of each CPC at different positions was determined. The membership functions of the nine CPCs were used to calculate the membership values, which can be used as the input values of the BN. Four experts were selected to score the nine CPC factors following the evaluation rules. The scores for CPCs of 13 operating positions were shown in S1 Table.

The fuzzy membership function was used to determine the linguistic variables in numerical forms. Therefore, the fuzzy set and corresponding membership degree were used to explain the linguistic variables. For example, the fuzzy set of organizational perfectness of CPC1 was deficient [a, b, c, d] = [0, 0, 10, 30],inefficient[a, b, c, d] = [10, 30, 30, 50], efficient [a, b, c, d] = [30, 50, 70, 80], and very effective [a, b, c, d] = [70, 80, 100, 100]. The fuzzy set of all CPCs were shown in S2 Table. The membership functions of the four control modes are shown in Formula (6).


$$\mu_{\left[a\;b\;c\;d\right]}\left(x\right)=\{\begin{array}{ll} 0 & \left(\text{x}\leq \text{a}\right)\\ \frac{x-a}{b-a} & \left(\text{a}\leq \text{x}\leq \text{b}\right)\\ 1 & \left(\text{b}\leq \text{x}\leq \text{c}\right)\\ \frac{d-x}{d-c} & \left(\text{c}\leq \text{x}\leq \text{d}\right)\\ 0 & \left(\text{x}\geq \text{d}\right) \end{array}$$ (5)

$$f(str)=(\begin{array}{cc} 1 & -5.3\leq x\leq -3\\ -x-2 & -3\leq x\leq -2\\ 0 & x\geq -2 \end{array}$$

$$f(tac)=(\begin{array}{cc} x+3 & -3\leq x\leq -2\\ -x-1 & -2\leq x\leq -1\\ 0 & x\leq -3,x\geq -1 \end{array}$$

$$f(opp)=(\begin{array}{cc} x+2 & -2\leq x\leq -1\\ \left(-x-0.3\right)/0.7 & -1\leq x\leq -0.3\\ 0 & x\leq -2,x\geq -0.3 \end{array}$$

$$f(scr)=(\begin{array}{cc} 0 & x\leq -1\\ x+1/0.7 & -1\leq x\leq -0.3\\ 1 & x=-0.3\\ -x/0.3 & -0.3\leq x\leq 0 \end{array}$$ (6)

### 4.3 Calculation of probability distribution for control modes and HEP

**Fig 4:**
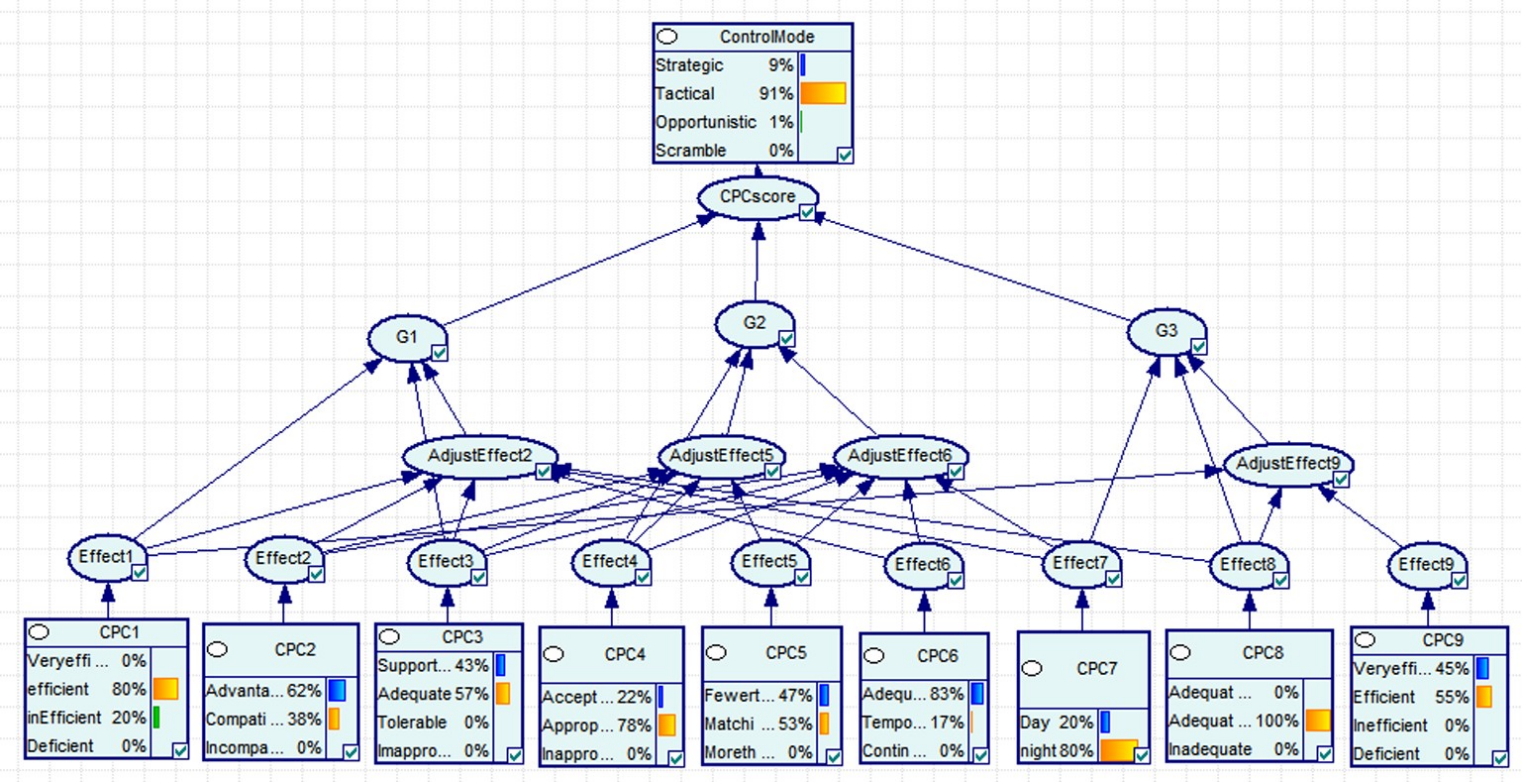
BN reasoning results for operation No. 1.

Among the 13 operating positions listed in our case study, the first operating position was taken as an example. After the evaluation of various factors of the first job position, it was converted to a BN input according to the membership function, as shown in Table 5. According to Fig 3, the probability distribution of each control mode was obtained. Subsequently, the probability distribution of each control mode of operation No. 1 was correspondingly 8.9% (strategic), 90.5% (tactical), 0.6% (opportunistic), and 0 (scrambled), as shown in Fig 4. According to Fig 2 and Eqs (2) and (3), using the COA technology, the exact value, namely the logarithm of HEP, was obtained by means of deambling analysis. Therefore, the probability of human mistake in the first job position was HEP_1_ = 10^−2.36861^ = 4.3×10^−3^.

**Table 5:** Input values of BN at different operating positions.

CPCs No.	CPC levels	(1)	(2)	(3)	(4)	(5)	(6)	(7)	(8)	(9)	(10)	(11)	(12)	(13)
CPC1	Veryefficient	0	0	0	0	0	0	0	0	0	0	0	0	0
	Efficient	0.8	0.705	0.888	0.41	0.459	0.425	1	1	0.8	1	0	0.195	1
	InEfficient	0.2	0.295	0.112	0.59	0.541	0.575	0	0	0.2	0	1	0.805	0
	Deficient	0	0	0	0	0	0	0	0	0	0	0	0	0
CPC2	Advantageous	0.615	0.45	0.265	0.104	0.11	0.02	0.524	0.775	0.645	0.735	0	0.005	0.715
	Compatible	0.385	0.55	0.735	0.896	0.89	0.98	0.476	0.225	0.355	0.265	1	0.995	0.285
	Incompatible	0	0	0	0	0	0	0	0	0	0	0	0	0
CPC3	Supportive	0.428	0	0	0	0	0	0.118	0.25	0.148	0	0	0	0.472
	Adequate	0.572	1	0.85	1	0.376	1	0.882	0.75	0.852	0.67	0	0	0.528
	Tolerable	0	0	0.15	0	0.624	0	0	0	0	0.33	0.1	0.898	0
	Imappropriate	0	0	0	0	0	0	0	0	0	0	0.9	0.102	0
CPC4	Acceptable	0.216	0	0.211	0	0	0	0	0	0.185	0	0	0	0.051
	Appropriate	0.784	0.899	0.789	0.475	0.91	0.485	0.985	0.827	0.815	0.92	0.295	0.405	0.949
	Inappropriate	0	0.101	0	0.525	0.09	0.515	0.015	0.173	0	0.08	0.705	0.595	0
CPC5	Fewer than capacity	0.47	0.586	0.396	0.177	0.241	0.045	0.625	0.84	0.635	0.595	0	0.213	0.59
	Matching current capacity	0.53	0.414	0.604	0.823	0.759	0.955	0.375	0.16	0.365	0.405	1	0.787	0.41
	More than capacity	0	0	0	0	0	0	0	0	0	0	0	0	0
CPC6	Adequate	0.831	0.2	0.465	0.285	0.291	0.19	0.712	0.854	0.687	0.84	0.41	0.375	1
	Temporarily inadequate	0.169	0.8	0.535	0.715	0.709	0.81	0.288	0.146	0.313	0.16	0.59	0.625	0
	Continuously inadequate	0	0	0	0	0	0	0	0	0	0	0	0	0
CPC7	Day	0.2	0.32	0.19	0.56	0.38	0.34	0.135	0.456	0.24	0.096	0.24	0.26	0.44
	night	0.8	0.68	0.81	0.44	0.62	0.66	0.865	0.544	0.76	0.904	0.76	0.74	0.56
	Night (yejian)	0	0	0	0	0	0	0	0	0	0	0	0	0
CPC8	Adequate high experience	0	0	0	0	0	0	0.142	0	0.276	0.06	0	0	0.22
	Adequate, limited experience	1	1	0.88	1	1	0.344	0.858	1	0.724	0.94	1	1	0.78
	Inadequate	0	0	0.12	0	0	0.656	0	0	0	0	0	0	0
CPC9	Very efficient	0.45	0	0	0	0	0	0.322	0.29	0.59	0.47	0	0	0.792
	Efficient	0.55	0.87	0.844	0.622	0.774	0	0.678	0.71	0.41	0.53	0	0	0.208
	Inefficient	0	0.13	0.156	0.378	0.226	1	0	0	0	0	1	1	0
	Deficient	0	0	0	0	0	0	0	0	0	0	0	0	0

### 4.4 Results and discussions

**Fig 5:**
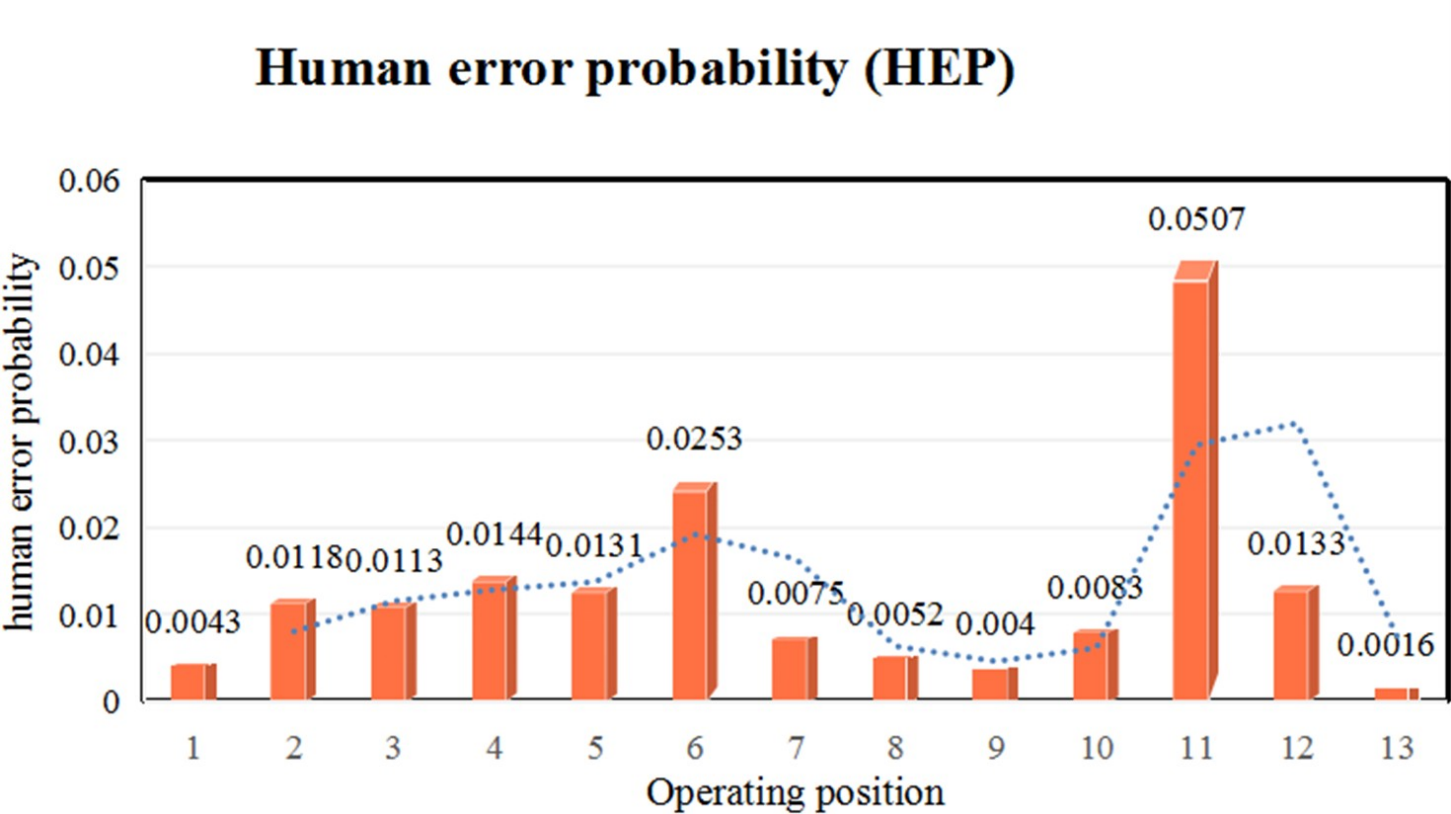
HEP curve at different positions.

Table 6 shows the probability distribution of different control modes in various working positions together with the probability of operator errors. Calculations showed that the control mode of operator in job position 11 was opportunistic, indicating that the probability of operator error was moderately high. To name the main reasons, the operator did not implement the standardized operational requirements (nonstandard operation), was in a wrong operation position, and the labour protection equipment was not fully worn. The rest of the study calculated the reliability of control mode for the operator of tactical, while control mode of the remaining job positions was Tactical, and Human error probability (HEP) ranking can be obtained at the same time. The job positions in the order of the error probability of the workers are (6), (4), (12), (5), (2), (3), (10), (7), (8), (1), (9), and (13), as shown in Fig 5. Analysing monthly human error times at different positions in an enterprise showed that the trend was basically identical, which proved that our proposed model was reasonable and had a certain practical significance.

**Table 6:** Control mode and reliability probability value of different operating positions.

Positions No.	Strategic	Tactical	Opportunistic	Scrambled	Dominant control mode	Crisp value	Human error probability (HEP)
(1)	0.089	0.905	0.006	0	Tactical	-2.36861	0.0043
(2)	0	0.943	0.057	0	Tactical	-1.92630	0.0118
(3)	0.001	0.956	0.043	0	Tactical	-1.94819	0.0113
(4)	0	0.861	0.139	0	Tactical	-1.84246	0.0144
(5)	0	0.904	0.096	0	Tactical	-1.88382	0.0131
(6)	0	0.504	0.496	0	Tactical	-1.59658	0.0253
(7)	0.025	0.974	0.001	0	Tactical	-2.12433	0.0075
(8)	0.062	0.937	0.001	0	Tactical	-2.28000	0.0052
(9)	0.097	0.896	0.007	0	Tactical	-2.39343	0.004
(10)	0.016	0.983	0.001	0	Tactical	-2.08128	0.0083
(11)	0	0.184	0.701	0.115	Opportunistic	-1.29503	0.0507
(12)	0	0.678	0.312	0.010	Tactical	-1.87703	0.0133
(13)	0.259	0.741	0	0	Tactical	-2.80234	0.0016

For comparison purposes, taking operation 1 and operation 11 as an example, the basic CREAM [29] are investigated (on the basis of the same input), as can be seen in Table 7, the results of the proposed quantified methodology are in the range of the expectations of probability intervals based on the basic CREAM. Therefore, the methodology proposed in this paper is validated to some extent. The improvement by the proposed methodology is that the output is probability evaluation with point values, which thus provides definitive values for quantitative risk assessment in the metallurgical industry.

**Table 7:** Comparison of the results from the basic CREAM with the results based on the quantified methodology.

Position No.	control mode		Human error probability (HEP)	
	according to the basic CREAM	according to the methodology pro posed in this research	Probability interval (according to the basic CREAM)	Human error Probability (according to the methodology proposed in this research)
(1)	Tactical	Tactical	0.001 < p < 0.1	4.3×10^−3^.
(11)	opportunistic	opportunistic	0.01 < p < 0.5	5.07×10^−2^.

**Fig 6:**
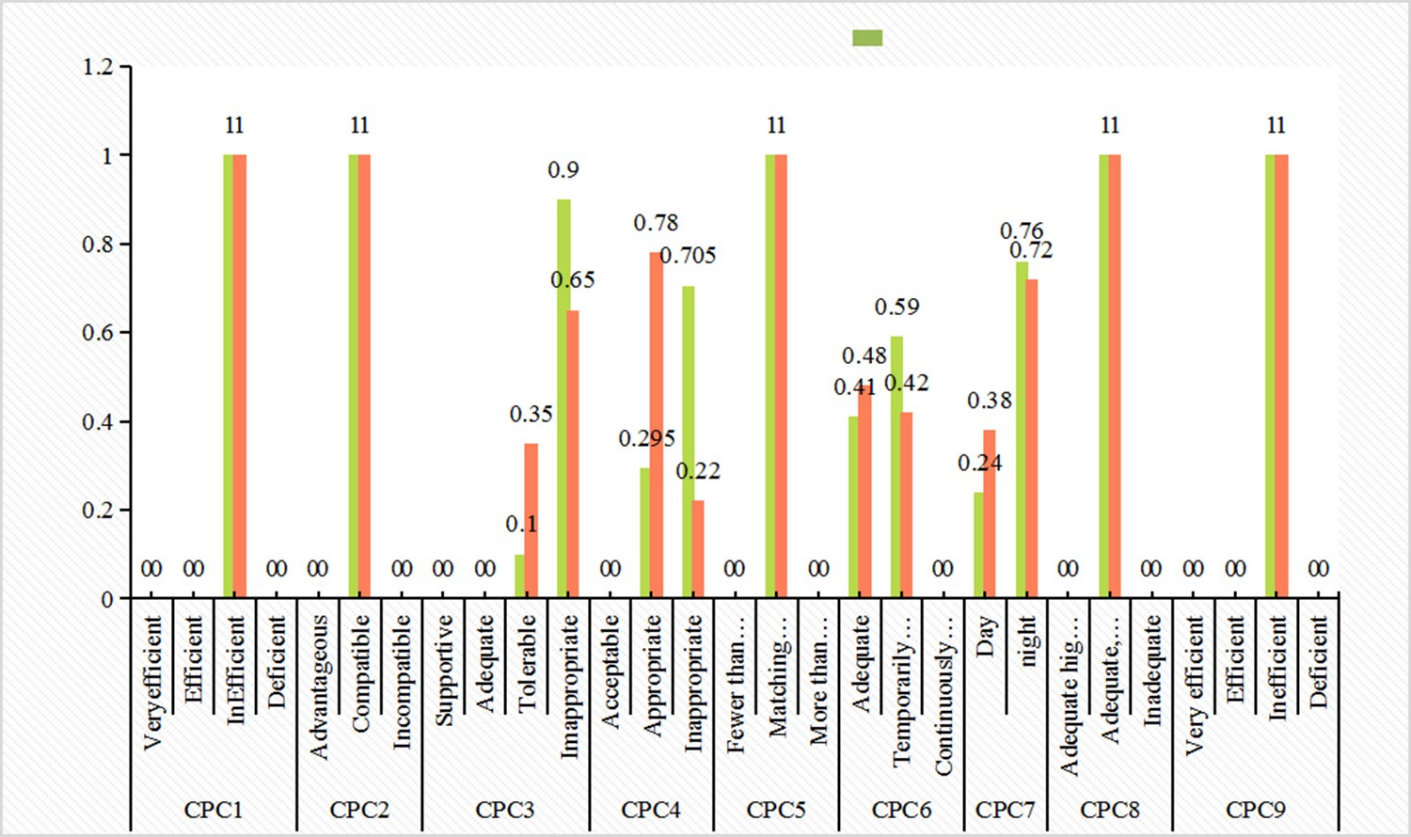
Position NO.11 BN diagnostic data diagram.

According to the established BN model, the human reliability of operation (11) was studied. When the control mode was set to ‘tactical’ as evidence, the corresponding effects of CPC3, CPC4, CPC6, and CPC7 had significant changes, and the most significant one was CPC4, as shown in Fig 6. This result showed the importance of operating procedures and emergency disposal requirements. To prevent and reduce the occurrence of accidents caused by human errors, operators can be trained to become acquainted with the operational procedures and emergency disposal requirements and pay attention to the effects of training. In addition, the training time for operators should also meet certain requirements.

By considering the daily task of operation number 11, the measures can include but not limited to the following aspects.

1. Carry out safety education, carry out training and assessment of operating skills/safe work practices, unqualified operators shall not work.
2. During operation, focus on monitoring the following unsafe behaviors:
  1. Operation without troubleshooting,
  2. Operating at an unsafe speed;
  3. Use unsafe equipment or use equipment unsafely;
  4. in an unsafe position or unsafe operating posture;
  5. Work in operation or on dangerous equipment.
  6. In the environment and places with occupational hazards, labor protection and products are not used or properly worn.
3. Set the prescribed safety warning signs and safety colors in the workplace or related equipment.
4. Publish emergency plans or measures at work posts, and organize regular drills.

Therefore, the results of this study provide deterministic values for quantitative risk assessments, such as event tree and fault tree in metallurgical enterprises. Additionally, it can be a reference for the safety training of personnel in different operating positions and for the classification of risk management and control operating positions.

## 5. Conclusions

Accidents inevitably occur in the smelting, transporting, and processing processes of high-temperature molten metal and slag in the metallurgical industry. All accidents are not only related to the complexity of the system but also to human error. Lack of reliable data in HRA has been a major problem in assessing the safety of personnel. CREAM has been broadly applied to analyse human reliability in many industries. This study extended the traditional CREAM method to a fuzzy environment to quantify human reliability probabilities. This aim was realized by incorporating the BN reasoning to model the dependency among CPCs. Considering the characteristics of high-temperature molten metal operation, the CPC evaluation terms of CREAM were refined. Therefore, they truly reflected the actual working characteristics and task environment.

The fuzzy logic theory was employed to establish the membership degree of CPCs, which was used as the input of the BN. The probability distribution of the control mode was obtained by BN reasoning and the reliability of different positions in high-temperature molten metal operation. Considering the HRA of 13 operators working in high-temperature molten metal operation, human reliability of the operators was obtained. Comparisons of the two aspects were done. Firstly, the results were compared to the accident/incident data. It may take years to collect enough accident data from the entire industry to conduct comparisons. Therefore, we only compared the accident data collected from a certain enterprise over the course of many years. The results showed that the proposed model could be applied in a wider range. Besides, the comparison between results of the basic CREAM and results of the proposed quantified methodology was done, the results of the proposed quantified methodology are in the range of the expectations of probability intervals based on the basic CREAM. So, the methodology proposed in this paper is validated.In addition, sensitivity factors were obtained by diagnosing the introduced model. Finally, establishing a safety management system can improve the safety training procedures and serve as a reference.

the contribution of this paper first proposes the assessment rules of the Common Performance Condition(CPC) suitable for high temperature molten metal operation, and then incorporates CREAM, fuzzy set theory and Bayesian network method to calculate the precise/point human error probability (instead of interval value) of different positions in high temperature molten metal for the first time. This can provide the precise human probability which can be used as input in the risk evaluation of the metallurgical industry.

In CREAM, it is considered that each CPC factor is equally important. Since this method originated in the field of nuclear industry, Theoretically, the original general efficiency conditions can not be fully applied to the characteristics of operations in the field of metallurgy industry [15, 17, 35]. Therefore, the CPC should assign different weights to different work natures. The next research will focus on the analysis of the model, and further improve the accuracy of the model.

## Supporting information

S1 Table The scores for CPCs of 13 operating positions. (DOCX)

S2 Table CPCs and their levels, and the fuzzy set. (DOCX)

S1 Appendix Questionnaire for CREAM evaluation. (DOCX)
