# Supplementary material for: Human reliability analysis of high-temperature molten metal operation based on fuzzy CREAM and Bayesian network
Source: PLoS One. 2021 Aug 2;16(8):e0254861. doi: 10.1371/journal.pone.0254861 (PMC8328327; doi:10.1371/journal.pone.0254861)
Supplement: S1 Table — (DOCX) [file pone.0254861.s001.docx]

**S1 Table.the scores for CPCs of 13operating positions**

| **CPCs No.** | **（1）** | **（2）** | **（3）** | **（4）** | **（5）** | **（6）** | **（7）** | **（8）** | **（9）** | **（10）** | **（11）** | **（12）** | **（13）** |
| --- | --- | --- | --- | --- | --- | --- | --- | --- | --- | --- | --- | --- | --- |
| **CPC1** | 46.00 | 44.10 | 47.76 | 38.20 | 39.18 | 38.50 | 50.00 | 50.00 | 46.00 | 50.00 | 30.00 | 33.90 | 50.00 |
| **CPC2** | 72.30 | 69.00 | 65.30 | 62.08 | 62.20 | 60.40 | 70.48 | 75.50 | 72.90 | 74.70 | 60.00 | 60.10 | 74.30 |
| **CPC3** | 74.28 | 70.00 | 58.50 | 70.00 | 53.76 | 70.00 | 71.18 | 72.50 | 71.48 | 56.70 | 41.00 | 48.98 | 74.72 |
| **CPC4** | 64.32 | 57.98 | 64.22 | 49.50 | 58.20 | 49.70 | 59.70 | 56.54 | 76.30 | 58.40 | 45.90 | 48.10 | 61.02 |
| **CPC5** | 69.40 | 71.72 | 67.92 | 63.54 | 64.82 | 60.90 | 72.50 | 76.80 | 72.70 | 71.90 | 60.00 | 64.26 | 71.80 |
| **CPC6** | 76.62 | 64.00 | 69.30 | 65.70 | 65.82 | 63.80 | 74.24 | 77.08 | 73.74 | 76.80 | 68.20 | 67.50 | 82.00 |
| **CPC7** | 61.00 | 61.60 | 60.95 | 62.80 | 61.90 | 61.70 | 60.68 | 62.28 | 61.20 | 60.48 | 61.20 | 61.30 | 62.20 |
| **CPC8** | 65.00 | 60.00 | 59.40 | 65.00 | 65.00 | 56.72 | 71.42 | 60.00 | 72.76 | 70.60 | 65.00 | 60.00 | 72.20 |
| **CPC9** | 74.50 | 58.70 | 58.44 | 56.22 | 57.74 | 50.00 | 73.22 | 72.90 | 75.90 | 74.70 | 50.00 | 50.00 | 77.92 |
