# Supplementary material for: Human reliability analysis of high-temperature molten metal operation based on fuzzy CREAM and Bayesian network
Source: PLoS One. 2021 Aug 2;16(8):e0254861. doi: 10.1371/journal.pone.0254861 (PMC8328327; doi:10.1371/journal.pone.0254861)
Supplement: S2 Table — (DOCX) [file pone.0254861.s002.docx]

**S2 Table. CPCs and their levels, and the fuzzy set**

| **CPCs No.** | | **CPCs** | **CPC levels** | **the fuzzy set** |
| --- | --- | --- | --- | --- |
| CPC1 | Adequacy of organisation | Veryefficient | [70, 80, 100, 100] |  |
|  |  | Efficient | [30, 50, 70, 80] |  |
|  |  | InEfficient | [10, 30, 30, 50] |  |
|  |  | Deficient | [0, 0, 10, 30] |  |
| CPC2 | Working condition | Advantageous | [60,80,100,100 ] |  |
|  |  | Compatible | [30,50,60,80 ] |  |
|  |  | Incompatible | [ 0,0,30,50 ] |  |
| CPC3 | Adequacy of man machine interface (MMI) and operational support | Supportive | [70,80,100,100 ] |  |
|  |  | Adequate | [50,60,70 ,80] |  |
|  |  | Tolerable | [40, 50,50 ,60] |  |
|  |  | Imappropriate | [ 0,0,40,50,] |  |
| CPC4 | Availability of procedures/plans | Acceptable | [60,80,100,100 ] |  |
|  |  | Appropriate | [40, 60,60 ,80] |  |
|  |  | Inappropriate | [0,0, 40, 60] |  |
| CPC5 | Number of simultaneous goals | Fewer than capacity | [60.80.100，100 ] |  |
|  |  | Matching current capacity | [40,60,60,80 ] |  |
|  |  | More than capacity | [0,0,40,60 ] |  |
| CPC6 | Available time | Adequate | [60,80,100,100 ] |  |
|  |  | Temporarily inadequate | [ 30，50，60，80] |  |
|  |  | Continuously inadequate | [0,0,30,50 ] |  |
| CPC7 | Time of day | Day | [60,65,100,100 ] |  |
|  |  | night | [30,40,60, 65] |  |
|  |  | Night (yejian ) | [0,0,30,40 ] |  |
| CPC8 | Adequacy of training and  expertise | Adequate high  experience | [70,80,100,100 ] |  |
|  |  | Adequate, limited  experience | [55,60,70,80 ] |  |
|  |  | Inadequate | [0,0,55,60 ] |  |
| CPC9 | Crew collaboration quality | Very efficient | [70,80,100,100 ] |  |
|  |  | Efficient | [ 50,60,70,80] |  |
|  |  | Inefficient | [20,30,50,60 ] |  |
|  |  | Deficient | [0,0,20,30 ] |  |
