# Supplementary material for: Human reliability analysis of high-temperature molten metal operation based on fuzzy CREAM and Bayesian network
Source: PLoS One. 2021 Aug 2;16(8):e0254861. doi: 10.1371/journal.pone.0254861 (PMC8328327; doi:10.1371/journal.pone.0254861)
Supplement: S1 Appendix — (DOCX) [file pone.0254861.s003.docx]

**S1 Appendix. Questionnaire for CREAM evaluation**

| **.CPC** | **Questions** |
| --- | --- |
| CPC1  Adequacy of organization | 1.1 Whether the safety management system is perfect  1.2 Whether the assignment of safety management responsibilities is clear  1.3 Whether employees are informed of danger  1.4 Are safety checks performed regularly |
| CPC2  Working condition | 1.1Are measures in place to mitigate high temperatures  1.2Whether the illumination of the working environment meets the requirements  1.3Are there noise protection measures in the working environment  1.4Considering the effect of thermal pressure, whether the working environment is ventilated |
| CPC3  Adequacy of Man Machine Interface (MMI) and operational support | 1.1 Whether the operation process instructions are clear  1.2 Whether the safety protection facilities meet the requirements  1.3Whether equipment and facilities are regularly inspected  1.4Is there a remote control system to provide operational support  1.5 Are safety signs clearly visible to everyone  1.6 Whether alarm and emergency shutdown measures can prevent the occurrence of failure consequences |
| CPC4  Availability of procedures/plans | 1.1 Are emergency response requirements clear  1.2 Whether there is a sound and feasible safe operation procedure |
| CPC5  Number of simultaneous goals | 1.1 Whether the job position is adequately staffed  1.2 Can operators improve their skills through regular training |
| CPC6  Available time | 1.1Whether the time available to complete the task is sufficient |
| CPC7  Time of day | 1.1Do you work in the evening  1.2Do you work during the day |
| CPC8  Adequacy of training and experience | 1.1Is there regular training  1.2 Is there a regular exchange of experience |
| CPC9  Crew collaboration quality | 1.1Whether the company has a good safety culture  1.2Does the person understand his/her job responsibilities |
